# Antagonism of Bcl-X_L_ is necessary for synergy between carboplatin and BH3 mimetics in ovarian cancer cells

**DOI:** 10.1186/s13048-016-0234-y

**Published:** 2016-04-14

**Authors:** Mohammed Najim Abed, Marwan Ibrahim Abdullah, Alan Richardson

**Affiliations:** Institute for Science and Technology in Medicine, Guy Hilton Research Centre, Keele University, Thornburrow Drive, Stoke-on-Trent, ST4 7QB UK; School of Pharmacy, Keele University, Keele, ST5 5BG UK

**Keywords:** Ovarian cancer, BH3 mimetics, Navitoclax, Venetoclax

## Abstract

**Background:**

BH3 mimetics are a class of drugs that antagonize the Bcl-2 family of apoptosis inhibitors. We have previously shown that these compounds can potentiate the activity of carboplatin against several ovarian cancer cell lines. However, recent clinical studies have highlighted that BH3 mimetics which antagonise Bcl-X_L_ are associated with significant thrombocytopenia. This has led to the development of ABT-199 which specifically inhibits Bcl-2. Unfortunately, Bcl-X_L_ appears to be more frequently deregulated in ovarian cancer than Bcl-2. We therefore compared the ability of ABT-199, and the Bcl-X_L_ selective compound WEHI-539, to potentiate the activity of carboplatin in ovarian cancer cell lines.

**Methods:**

WEHI-539, ABT-737 and ABT-199 were tested in combination with carboplatin using a panel of 6 ovarian cancer cell lines. The activity of the drugs was evaluated using cell growth assays, staining with trypan bue and measurement of apoptosis by measuring caspase 3/7 activity, PARP cleavage and annexin-V/propidium iodide staining.

**Results:**

We found that WEHI-539 and ABT-737, but not ABT-199, were synergistic with carboplatin in cell growth assays and potentiated cell death when assessed by trypan blue staining. Furthermore, WEHI-539 and ABT-737 augmented carboplatin induced caspase 3/7 activity, PARP cleavage and annexin V labelling, but ABT-199 failed to do so.

**Conclusions:**

These observations suggest that compounds which target Bcl-X_L_ are necessary if BH3 mimetics are to be successfully used to treat patients with ovarian cancer and this highlights the need to develop strategies to minimize thrombocytopenia induced by such compounds.

## Background

Ovarian cancer (OC) is a heterogeneous disease characterized by low incidence, affecting around 4 % of women, but with rapid progression and high mortality rate [1]. Although many strategies have been developed to improve the treatment of OC, it is still the fifth leading cause of death in females with cancer. Patients with OC are often diagnosed late in the course of the disease because the symptoms are subtle and women frequently remain unaware of the disease until it reaches advanced stages [2]. The standard treatment of OC involves cytoreductive surgery followed by platinum-based combination therapy. Although most patients respond to this therapy, the development of chemoresistance prevents long-lasting treatment for OC patients and only 40 % of patients survive 5 years after diagnosis with advanced disease [3, 4]. Advances in understanding of the molecular basis of chemoresistance and inefficient apoptosis are of great importance for the development of targetted therapeutic approaches that might lead to better outcomes than conventional methods alone [5].

One of the major causes for the development of drug resistance is faulty apoptosis, one cause of which is overexpression of anti-apoptotic members of Bcl-2 family [6]. The contribution of Bcl-2 family proteins to the emergence of drug resistance has made them attractive targets for the development of new therapies to treat OC. The intrinsic apoptosis pathway is regulated by the Bcl-2 family of proteins. Bcl-2, Bcl-X_L_, Bcl-W, Mcl-1, and Bcl-2A1 act as inhibitors of this pathway by sequestering other pro-apoptotic family members [7–9]. BH3-mimetics are a class of compound that bind to the apoptosis inhibitors, preventing them from binding the pro-apoptotic proteins and thereby potentiating apoptosis [10]. In addition to overcoming drug resistance by promoting apoptosis, BH3 mimetics also induce autophagy. This is mediated through several mechanisms, including the liberation of the autophagy regulator Beclin from Bcl-2 family proteins [11]. Autophagy has been linked to both cell survival and cell death and so BH3 mimetics may also modulate the effect of cytotoxic agents through this pathway. The most prominent drugs in this class are ABT-737, and its closely related orally bioavailable counterpart navitoclax (ABT-263). Both of these compounds can inhibit Bcl-2, Bcl-X_L_ and Bcl-W but not Mcl-1 [12, 13]. We have shown that both these compounds can potentiate apoptosis induced by carboplatin using in vitro and xenograft models of ovarian cancer [14, 15]. Although navitoclax has progressed to clinical trials and there have been initial signs of efficacy in some cancers, navitoclax also produced dose dependent thrombocytopenia by antagonizing the survival function of Bcl-X_L_ in platelets [16]. As a result of this, ABT-199 (venetoclax) was developed by re-engineering navitoclax to produce a drug which selectively inhibits Bcl-2 protein but not Bcl-X_L_. Clinical studies have demonstrated that ABT-199 does not cause significant thrombocytopenia and its efficacy is currently being evaluated in a number of cancer types [17–19]. This led us to consider whether ABT-199 would also be effective in ovarian cancer. In our initial studies we noted that Bcl-2 is not widely expressed in ovarian cancer cell lines and this has also been observed in clinical samples [20]. This is also confirmed by interrogation of the cancer genome atlas which reports Bcl-2 is amplified or mRNA upregulated in less than 3 % of cases [21]. In contrast, the proportion of cases in which amplification or mRNA upregulation of Bcl-X_L_ (14 %), Bcl-W (12 %), or Mcl-1 (14 %) is observed is notably higher. This led us to question whether a Bcl-2 selective inhibitor would be of therapeutic use in a significant proportion of ovarian cancer patients. Instead, we hypothesized that a Bcl-X_L_ selective inhibitor would be preferable, although additional strategies would be necessary to overcome the likely ensuing thrombocytopenia. WEHI-539 is a recently described selective inhibitor of Bcl-X_L_. We therefore compared the ability of ABT-199 and WEHI-539 to potentiate the activity of carboplatin. ABT-737, which antagonises Bcl-2, Bcl-X_L_ and Bcl-2, was included as a comparator. In several models, we found that WEHI-539, as well as ABT-737, augmented the activity of carboplatin but ABT-199 failed to do so. These observations suggest that compounds targeting Bcl-X_L_ will be of benefit in ovarian cancer, but novel strategies to minimize thrombocytopenia will be necessary.

## Methods

Ovcar-8, Ovcar-3, Ovcar-4 and Ovsaho cells were grown in the RPMI, Igrov-1, Cov-362 and Cov-318 cells were grown in DMEM and Fuov-1 cells were grown in DMEM/F-12 nutrient mixture. Growth medium were supplemented with 10 % FCS, L-Glutamine (2 mM) and penicillin-streptomycin (50 IU/ml). For Ovcar-3, cells the medium was further supplemented with Insulin (0.01 mg/ml) and sodium pyruvate (0.11 g/l). ABT-737, ABT-199 and WEHI-539 (Medchem Express, NJ, USA), were prepared as a 20 mmol/L solution in dimethyl sulfoxide, whereas the stock solution of carboplatin was prepared in phosphate-buffered saline (PBS) at 13.5 mmol/L.

For cell growth assays, cells were plated in 96 wells plate (5,000 cells/well for all cell lines except Ovcar-8 which was plated at a density of 2,500 cells/well). The next day, cells were treated with drugs. After 72 h the culture medium was removed and the cells were fixed with 100 μl of cold 10 % Trichloroacetic acid (TCA), incubated on ice for 30 min and stained with 0.4 % sulforhodamine B (SRB) as described [14]. The data were analysed by using Graphpad Prism 4 software. Non-linear regression was used to fit a four parameters Hill equation. For drug combinations studies the cells were exposed simultaneously to a range of concentrations of carboplatin combined with fixed concentration of BH3 mimetics that was expected from the single agent studies to cause 5 % growth inhibition: ABT-737, 1 μM in Ovcar-8, Ovcar-3 and Igrov-1, 2 μM in Ovcar-4 and Ovsaho and 6 μM in Cov-362; ABT-199, 1 μM in Ovcar-4, 2 μM in Ovcar-3, Igrov-1, Cov-362 and Ovsaho and 3 μM in Ovcar-8; WEHI-539, 0.2 μM in Igrov-1, 0.3 μM in Ovcar-8, 1 μM in Ovcar-3 and Ovsaho, 3.1 μM in Cov-362 and 5 μM in Ovcar-4. Surviving cell number was assessed by SRB staining as describe above. A combination index (CI) was calculated as described by using Chou and Talalay [22, 23].

To assess viability using trypan blue, cells were plated in 12 well plates and exposed to the indicated drug concentration for 48 h except for Ovcar-4 which was assessed after 24 h. The supernatant was collected and adherent cells detached with trypsin. The two samples were combined, and cells collected by centrifugation (150 × g, 3 min) before re-suspending in 0.2 % trypan blue in PBS and counting live and dead cells on a haemocytometer.

To quantify apoptosis, cells were plated in 96 well plates and exposed to the indicated concentration of carboplatin and/or BH3 mimetic as described above. After 48 h, 25 μl of Caspase 3/7 Glo-reagent (Promega) was added and luminescence measured after 30 min. The Bliss independence criterion [24, 25] was used to calculate the expected effect of drug combinations.

$$ \mathrm{E}\ \left(\mathrm{x},\mathrm{y}\right) = \mathrm{E}\ \left(\mathrm{x}\right) + \mathrm{E}\ \left(\mathrm{y}\right)\ \hbox{--}\ \mathrm{E}\ \left(\mathrm{x}\right)\ *\ \mathrm{E}\ \left(\mathrm{y}\right) $$

Where E is the expected effect of the combination and E(x) and E(y) are the effect of individual drugs.

To measure PARP cleavage, cells were seeded in 12 wells plate and treated with drugs and collected as described above. After washing with PBS, cells pellets were lysed with RIPA buffer (3 ml) comprising 20 mM Hepes, 150 mM NaCl, 2 mM ethylenediamine tetra-acetic acid, 0.5 % sodium deoxycholate, 1 % Nonidet-P40, and supplemented with 0.12 mM leupeptin, 0.05 mM pepstatin and 1 mM phenylmethylsulfonyl fluoride. Proteins concentration was estimated with a bicinchoninic acid (BCA) assay. Samples (10 μg) were analysed on 4-20 % Hepes gels (NuSep), electro-transferred to polyvinyldifluoride and analysed with PARP antibody (Cell Signalling Technology #9541, 1:2000).

To measure annexin V labelling, cells were seeded in 12 well plates, exposed to the indicated drug concentration for 48 h. The cells were labelled using a Annexin V-FITC kit (Miltenyi biotech) according to the manufactur’s instructions and analysed by flow cytometry.

## Results

**Fig. 1:**
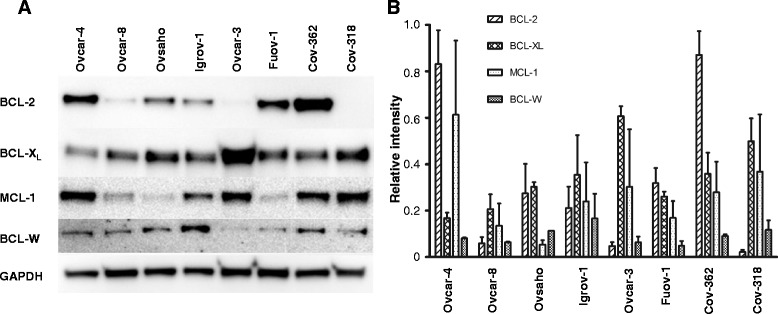
**a**. Expression of Bcl-2 family members. Lysates were prepared from the indicated cell lines and the expression of Bcl-2 family proteins determined by immunoblotting. The experiment shown is representative of 3 experiments. **b**. The western blots shown in Fig. 1a were quantified by normalizing the signal intensity of each band to that of GAPDH in the cell line and in the same blot (mean ± S.D., *n* = 3)

Prior to evaluating drug combinations of carboplatin and the BH3 mimetics, the expression of Bcl-2 family members and the single agent activity of ABT-737, ABT-199 and WEHI-539 in cell growth assays were first determined. We measured the expression of Bcl2 family members in 8 ovarian cancer cell lines (Fig. 1), This included three ovarian cancer cell lines Ovcar-3, Ovcar-8 and Igrov-1 in which we have previously observed synergy between carboplatin and ABT-737. These were supplemented with 5 additional cell lines that have recently been shown to closely resemble the profile of high grade serous ovarian cancer [26]. Bcl-X_L_ was expressed in all 8 cell lines. Bcl-2 expression was more variable, although it was detectable in 5 lines it was only prominent in 3 cell lines. Mcl-1 expression was also notable in 5 cell lines. In cell growth assays, all three compounds inhibited the growth of cultures of each cell line with microMolar potencies (Table 1).

**Table 1:** Activity of ABT-737, ABT-199 and WEHI-539 in ovarian cancer cell lines cell growth assays. Cells were treated with the indicated BH3 mimetic for 72 h and surviving cell number estimated by staining with SRB. The results are expressed as mean ± S.D. of the number of experiments shown in parentheses

IC_50_ (μM)			
Cell line	ABT-737	ABT-199	WEHI-539
OVCAR-8	11 ± 5 (12)	18 ± 6 (13)	9 ± 5 (11)
OVCAR-3	10 ± 4 (11)	15 ± 5 (12)	9 ± 4 (14)
IGROV-1	11 ± 1 (12)	14 ± 8 (14)	16 ± 12 (12)
COV-362	17 ± 3 (10)	4 ± 2 (9)	12 ± 2 (9)
OVCAR-4	14 ± 5 (8)	15 ± 2 (7)	26 ± 9 (7)
OVSAHO	7 ± 1 (10)	17 ± 9 (6)	14 ± 1 (6)
COV-318	20 ± 3 (5)	24 ± 2 (3)	23 ± 5 (3)
FUOV-1	13 ± 1 (4)	14 ± 6 (3)	27 ± 4 (3)

**Fig. 2:**
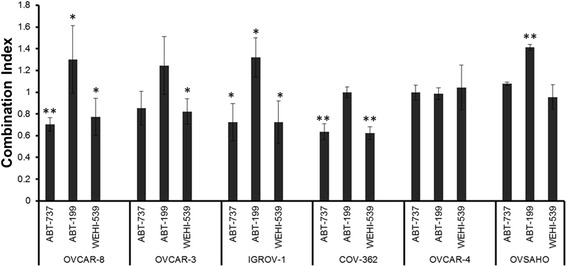
Evaluation of drug combinations in cell growth assays. The indicated cells were treated with combinations of carboplatin and the indicated BH3 mimetic. After 72 h, the surviving cell number was estimated by staining with SRB. Combination indices (mean ± S.D., *n* = 3) at fraction affected = 0.5 were significantly different from CI = 1.0 where indicated (paired t-test, *P ≤ 0.05 and **P ≤ 0.01.)

To evaluate which of the BH3 mimetics was synergistic with carboplatin, drug combination studies were performed in 6 cell lines using a fixed concentration of each BH3 mimetic and a range of concentrations of carboplatin. ABT-737 (which inhibits Bcl-2 as well as Bcl-X_L_) was synergistic with carboplatin in three cell lines (Fig. 2); this included Igrov-1 and Ovcar-8, cells in which we have previously observed synergy [14] as well as Cov-362. We have also previously reported synergy in Ovcar-3 cells [14] and the results obtained here were not inconsistent with that but did not reach statistical significance. The Bcl-X_L_ selective compound WEHI-539 was also synergistic with carboplatin in these cell lines. In Ovcar-4 and Ovsaho, synergy between ABT-737 and carboplatin was not observed. In contrast, the Bcl-2 selective compound ABT-199 was not synergistic with carboplatin in any of the cell lines and was even antagonistic with carboplatin in Ovcar-8, Igrov-1 and Ovsaho cells.

**Fig. 3:**
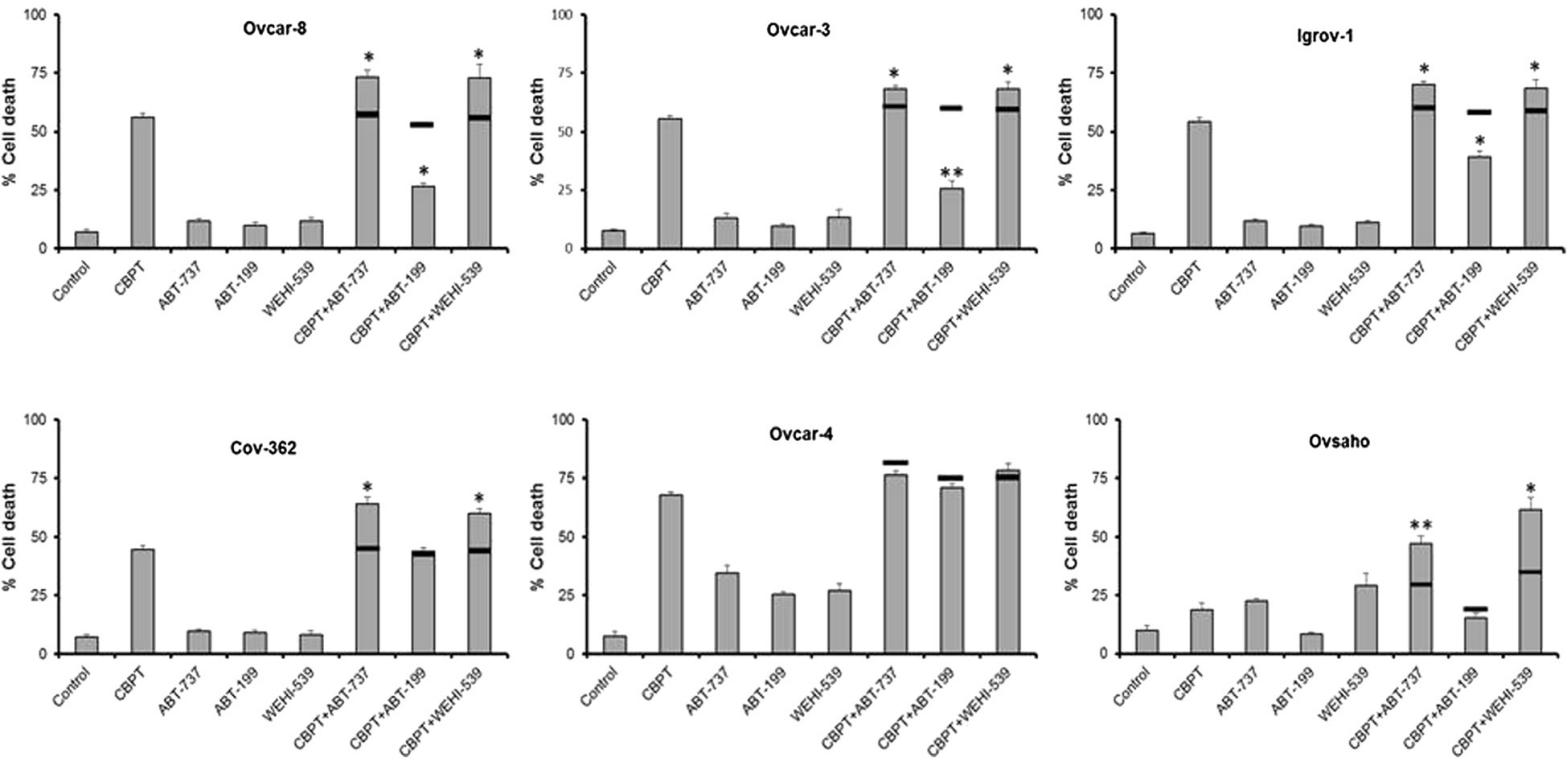
The effect of BH3 mimetic combinations on cell viability. The indicated cells were treated for 48 h (24 h for Ovcar-4) with carboplatin (66 μM), the indicated BH3 mimetic (concentraions are described in Methods) or a combination of the drugs as shown. Cells were stained with trypan blue and the percentage of dead cells determined by microscopy. Data are expressed as the fraction of viable cells measured in cells treated with drug vehicle (mean ± SD, *n* = 3). The horizontal solid lines on the bar chart indicate the expected effect of the combination calculated using the Bliss independent effect and the effect of the single agents. Experimental results are significantly different from Bliss expected effect where indicated (*P ≤ 0.05 and **P ≤ 0.01, paired t-test)

To confirm these results, the effects of the drug combinations on cell viability were assessed using trypan blue staining (Fig. 3). Consistent with the results obtained in the cell growth assays with Ovcar-8, Igrov-1 and Cov-362 cells, as well as with Ovcar-3 cells, combinations of carboplatin and ABT-737 or WEHI-539 killed significantly more than the expected effect of the combination calculated from the Bliss independence criterion and the effect of the single agents. We also observed supra-additive effects of carboplatin and ABT-737 or WEHI-539 in Ovsaho cells, despite the fact we did not observe synergy in these cells in cell growth assays. Notably, Ovsaho cells express a relatively high level of Bcl-X_L_ compared to Mcl-1 (Fig. 1). In contrast, supra-additive effects were not observed with ABT-199 and carboplatin in any of the cell lines and in 3 cell lines, (Ovcar-8, Igrov-1 and Ovsaho) the ABT-199 and carboplatin drug combination were antagonistic because significantly fewer dead cells were observed than expected.

**Fig. 4:**
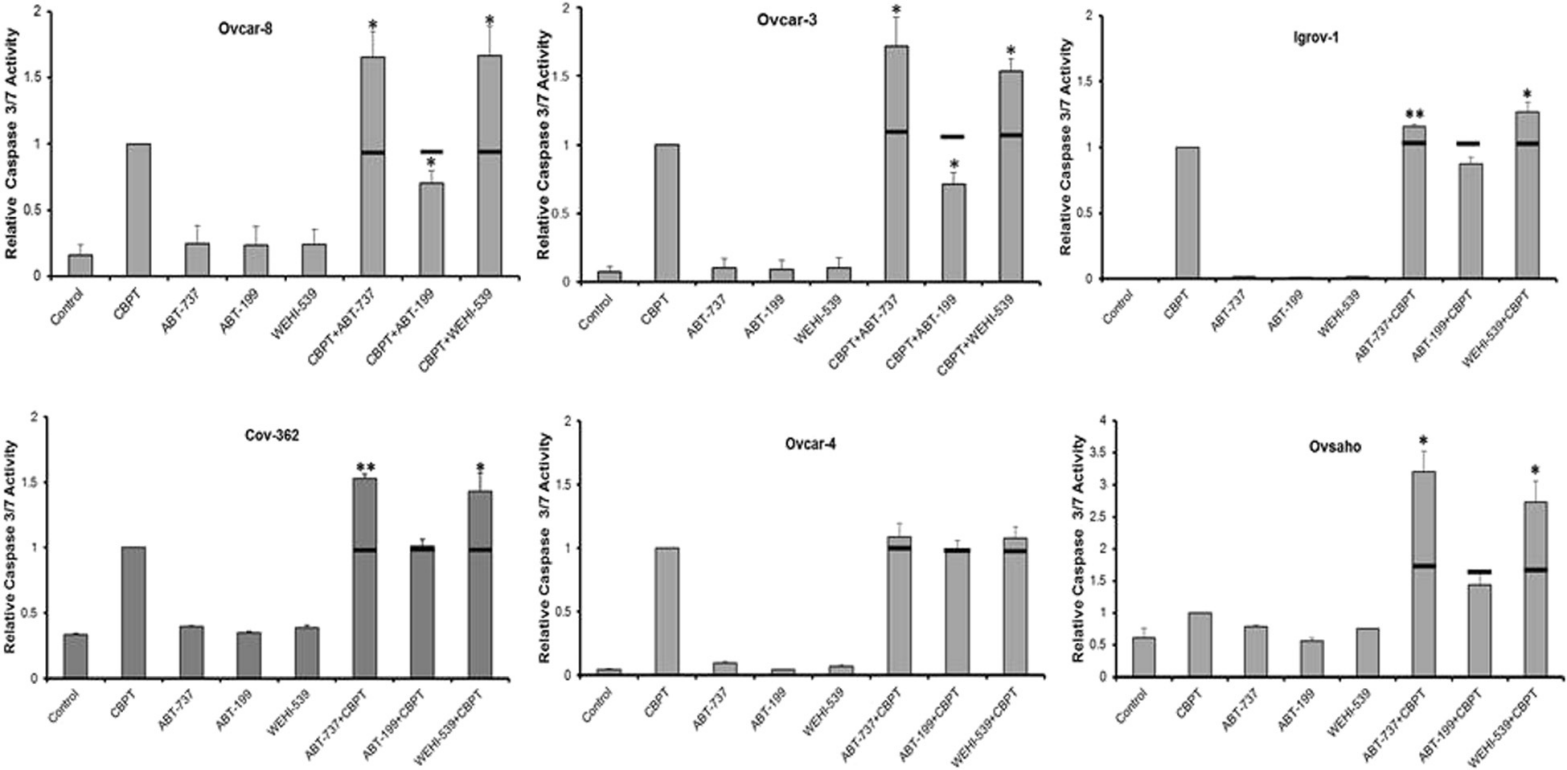
The effect of BH3 mimetic combinations on caspase 3/7 activity. The indicated cells were exposed to 66 μM of carboplatin in all cell lines or the indicated BH3 mimetic (as described in Methods) for 48 h (24 h for Ovcar-4) and the caspase activity then measured. The results (mean ± S.D., *n* = 3) were expressed as a fraction of the caspase activity measured in cells treated with carboplatin alone and were normalized to surviving cell number estimated by staining with SRB. The results were significantly different where indicated (*, *P* < 0.05, paired t-test) from the expected effect of the drug combination indicated with an horizontal solid line and calculated from the Bliss independence criterion and the effect of the single agents

In addition to the effect in cell growth assays, we have previously shown that ABT-737 and navitoclax potentiate apoptosis induced by carboplatin. To investigate the effect of the selective BH3 mimetics we measured caspase activity, PARP cleavage and Annexin V/Propidium iodide staining. The activation of caspase 3/7 by the drug combinations (Fig. 4) was reminiscent of the activity fo the cells in the foregoing studies. In 5 of the 6 of the cell lines, the combinations of carboplatin with either ABT-737 or WEHI-539 increased caspase-3/7 activity insignificantly more than the combined effect expected from applying the Bliss indepedance criterion to the effect of the single agents. In Ovcar-4 cells, cells which expressed relatively low levels of Bcl-X_L_ (Fig. 1) and in which synergy was not observed in cell growth assays (Fig. 2), the effect on caspase-3/7 activity of combinations of ABT-737 or WEHI-539 with carboplatin did not differ significantly from additivity. In contrast to ABT-737 and WEHI-539, synergistic effects were not observed with combinations of ABT-199 and carboplatin in any of the cells and antagonistic effects were observed in Ovcar-8 and Ovcar-3 cells.

**Fig. 5:**
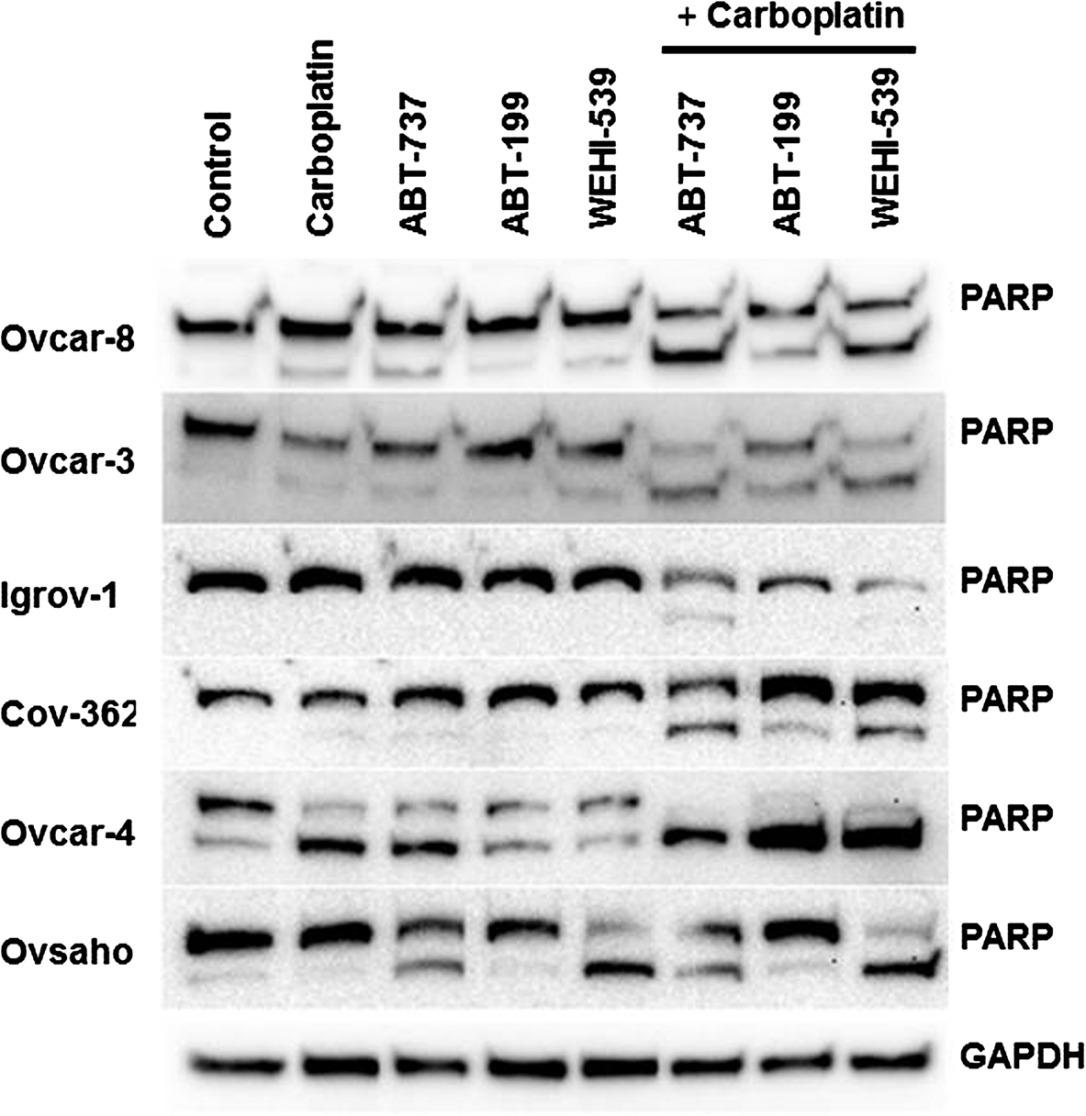
The effect of BH3 mimetic combinations on PARP cleavage . The indicated cells were exposed to 66 μM carboplatin or the indicated BH3 mimetic for 48 h (doses as indicated in Methods) and PARP cleavage assessed by immunoblotting. The results are representative of 3 experiments

In Ovcar-3, Ovcar-8, Igrov and Cov-362 cells, the BH3 mimetics did not induce noticeable PARP cleavage when they were tested as single agents (Fig. 5). As a single agent ABT-199 only induced PARP cleavage in Ovcar-4 cells and it is noteable that these cells expressed the least amount of Bcl-X_L_ (Fig. 1). ABT-737 and WEHI-539 as single agents caused noticeable PARP cleavage in Ovcar-4 and Ovsaho cells and noteably, Ovsaho cells expressed a relatively high ratio of Bcl-X_L_ compared to MCl-1 (Fig. 1). When used in combination, ABT-737 and WEHI-539 potentiated PARP cleavage induced by carboplatin; this was most prominent in the cells where synergy with carboplatin was observed in cell growth, trypan blue or caspase assays. In Ovcar-4 cells, the combination of carboplatin and ABT-737 induced very significant PARP cleavage, but in these cells the single agents also caused significant cleavage (Fig. 5). In contrast to ABT-737 and WEHI, ABT-199 failed to augment carboplatin-induced PARP cleavage.

**Fig. 6:**
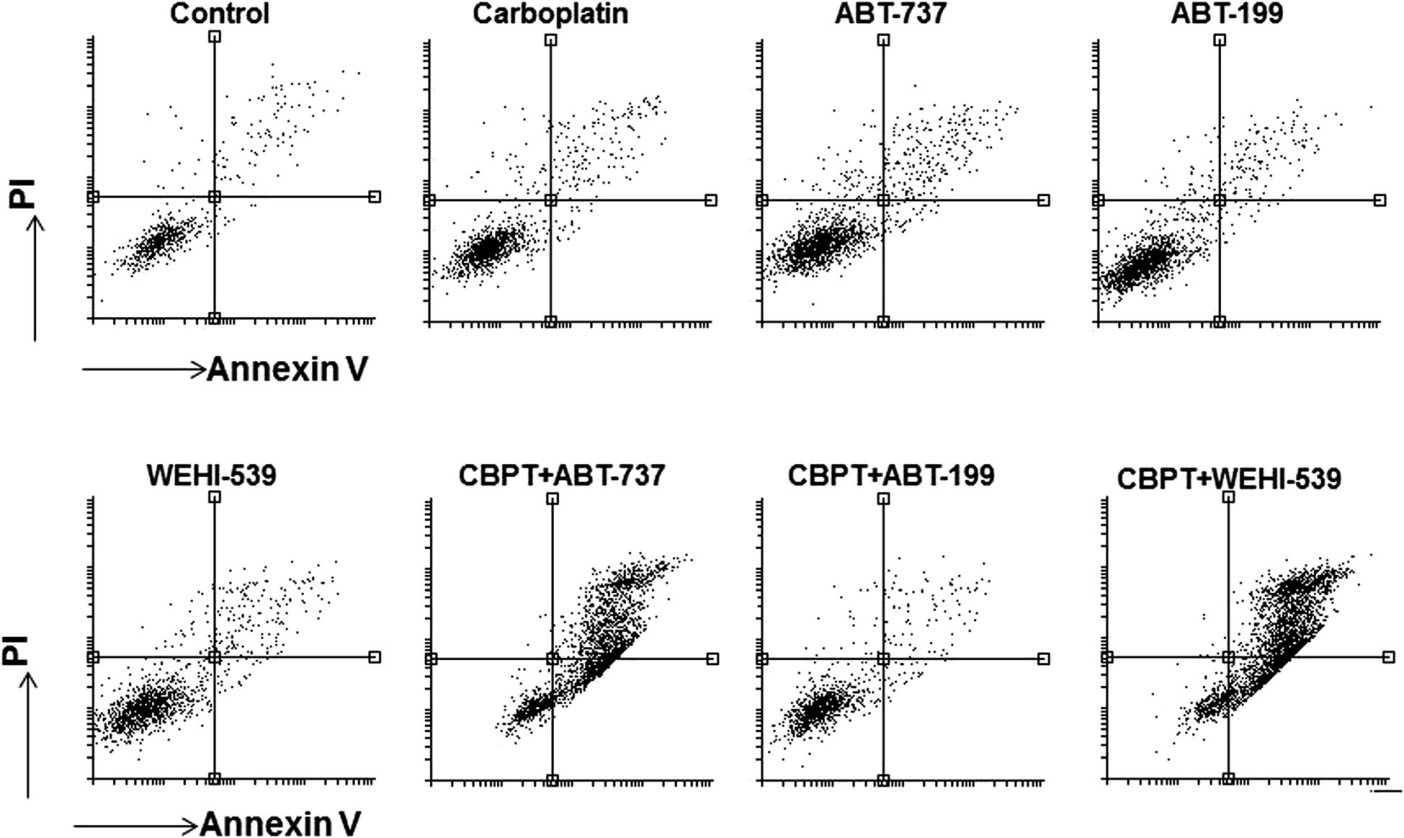
The effect of BH3 mimetic combinations on annexin V/propidium iodide staining. Ovcar-8 cells were exposed to 66 μM carboplatin or the indicated BH3 mimetic (at the concentration indicated in the Methods section) for 48 h, the cells were labelled with annexin V and propidium iodide and assessed by flow cytometery. The results shown are representative of 3 experiments. The results with other cell lines are summarized in Table 2

Flow cytometry experiments assessing annexin V/propidium iodide staining (Fig. 6 and Table 2) showed that BH3 mimetics induced apoptosis in a manner consistent with the caspase and PARP cleavage assays. ABT-737 and WEHI-539 combined with carboplatin induced supra-additive staining in Ovcar3, Ovcar-8, Igrov and Cov-326 cells, whereas the effect of ABT-199 and carboplatin combinations did not differ significantly from that expected if the drugs acted additively.

**Table 2:** The effect of BH3 mimetic combinations on annexin V/propidium iodide staining

	Annexin V/PI positive cells (% of total)					
	OVCAR-8	OVCAR-3	IGROV-1	COV-362	OVCAR-4	OVSAHO
Control	3 ± 1	4 ± 2	5 ± 1	2 ± 1	5 ± 2	2 ± 1
Carboplatin	11 ± 2	10 ± 3	15 ± 5	3 ± 1	75 ± 9	3 ± 2
ABT-737	6 ± 2	8 ± 1	5 ± 1	2 ± 1	17 ± 11	5 ± 1
ABT-199	4 ± 2	4 ± 1	5 ± 2	2 ± 1	8 ± 6	2 ± 1
WEHI-539	5 ± 2	6 ± 2	12 ± 5	2 ± 1	13 ± 9	6 ± 1
CbPt + ABT-737	72 ± 3**	58 ± 4*	39 ± 7	17 ± 5**	88 ± 4	4 ± 1
CbPt + ABT-199	13 ± 1	12 ± 7	16 ± 4	4 ± 1	84 ± 3	2 ± 1
CbPt + WEHI-539	79 ± 2**	71 ± 10*	35 ± 6	14 ± 2**	88 ± 5	5 ± 2

The indicated cells were exposed to 66 μM carboplatin or the indicated BH3 mimetic for 48 h, the cells were labelled with annexin V and propidium iodide and assessed by flow cytometery. The percentage of cells that stained positive with both annexin V and propidium iodide is shown. This was significantly different where shown paired t-test, *, *P* < 0.05; **, *P* < 0.01) from the percentage of annexinV/propidium iodide positive cells expected from the activity of the single agents and calculated from the Bliss independence criterion

## Discussion

Dysregulation of apoptosis is regarded as one of the major contributors to drug resistance, which may result from overexpression of the anti-apoptotic Bcl-2 proteins [27, 28]. Targeting these proteins with drugs that can effectively bind to and inactivate them provides a promising strategy for improving the quality of treatment, especially when combined with standard anti-cancer drugs [26]. We have previously shown that ABT-737 and navitoclax, BH3 mimetics which can inhibit both Bcl-2 and Bcl-X_L,_ potentiate the activity of carboplatin in ovarian cancer cell lines [14, 15]. In this study, we have compared the ability of the Bcl-2 and Bcl-X_L_ selective compounds ABT-199 and WEHI-539 to potentiate the activity of carboplatin. WEHI-539 was synergistic with carboplatin in several cell lines using several assays including cell growth, cell death, activation of caspase 3/7, PARP cleavage and Annexin V/propidium iodide staining. In contrast, in the same set of assays, ABT-199 did not potentiate the cell death induced by carboplatin and was even mildly antagonistic with carboplatin in some assays.

The observation that ABT-199 is antagonistic with carboplatin in several ovarian cancer cell lines is concerning because this compound is undergoing clinical evaluation as an alternative to navitoclax, a BH3 mimetic pharmacologically similar to ABT-737 and which causes thrombocytopenia. The reason for antagonism between carboplatin and ABT-199 that was observed in several cell lines is unclear. Antagonism was most evident in Ovcar-3, Ovcar-8 and Igrov-1 cells which express relatively low amounts of Bcl-2. Additivity was observed between carboplatin and ABT-199 and carboplatin in Ovcar-4 cells and COV-362 cells which expressed higher levels of Bcl-2. Bcl-2 has been linked to regulation of the cell cycle and DNA damage responses [29, 30] and this may contribute to the antagonism with carboplatin. Alternatively, this may represent inhibition by ABT-199 of a target other than Bcl-2. Whatever the mechanism, any clinical evaluation of ABT-199 and carboplatin in combination should only be pursued with caution.

Synergy between ABT-737 or WEHI-539 and carboplatin was observed in several cell lines using several different assays. The pre-eminent exception to this was Ovcar-4 cells, where the combinations of these were additive in all the cell lines we tested. It is noteable that, of all the cell lines evaluated, Ovcar-4 express the lowerst level of Bcl-X_L_, which is inhibited by both ABT-737 and WEHI-539. It is also noteable that in this cell line ABT-737 and WEHI-539 induced apoptosis when they were tested as single agents. Thus, the lack of synergy between carboplatin and these compounds in Ovcar-4 cells may reflect the low level of Bcl-X_L_ expression which allows these BH3 mimetics to induce apoptosis on their own, so synergy is not readily observed.

Our data strongly support the argument that drugs which inhibit Bcl-X_L_ will be necessary if BH3 mimetics are to be successfully used in the treatment of ovarian cancer. This is reinforced by the more frequent deregulation of Bcl-X_L_ than Bcl-2 in ovarian cancer reported in the cancer genome atlas [21]. Consequently, we consider that the Bcl-2 selective compound ABT-199 is unlikely to be efficacious in most ovarian cancers. However, it may be useful in the small fraction of ovarian cancer patients that express high levels of Bcl-2 [21]. A personalized medicine approach is likely to be necessary to use ABT-199 in ovarian cancer and measurement of Bcl-2 expression in patients’ tumours seems advisable, particularly in light of the antagonism we have observed in some cell lines. Navitoclax, which has undergone clinical evaluation in several cancers, inhibits Bcl-2, Bcl-X_L_ and Bcl-w and consequently it is likely to be effective in potentiating the activity of carboplatin in a larger proportion of ovarian cancer patients than ABT-199. However, navitoclax has been closely linked to thrombocytopenia. The same may be anticipated with WEHI-539 because both drugs inhibit Bcl-X_L_ that promotes platelet survival. A therapeutic window may be found by careful dose escalation studies, Alternatively, novel strategies may be necessary to minimize this adverse effect. For example, encapsulation of ABT-737 in nanoparticles has been shown to enhance its anti-tumour effects while minimizing thrombocytopenia [31]. Alternatively, we have shown more than additive effects when paclitaxel, which is known to have “platelet sparing” properties, is combined with navitoclax and carboplatin [15]. This may create an adequate therapeutic window to allow the use of navitoclax in ovarian cancer.

## Conclusion

We conclude that navitoclax remains the preferred BH3 mimetic for the treatment of ovarian cancer, but strategies to minimize thrombocytopenia will be essential if this drug is to be used safely.

### Availability of data

The datasets supporting the conclusions of this article are included within the article.

## Abbreviations

BH3: Bcl-2 homology 3
OC: ovarian cancer
PARP: poly(ADP-ribose) polymerase
PBS: phosphate-buffered saline
SRB: sulforhodamine B
TCA: trichloroacetic acid
